# Numerical Analysis of the Mechanical Response of Two-Phase Nanocomposites Consisting of Nanoporous Gold and Polymer

**DOI:** 10.3390/ma15041574

**Published:** 2022-02-19

**Authors:** Aleksandr Shalimov, Mikhail Tashkinov

**Affiliations:** Laboratory of Mechanics of Biocompatible Materials and Devices, Perm National Research Polytechnic University, 614990 Perm, Russia; shalimov96@pstu.ru

**Keywords:** bicontinuous structures, representative volume element, finite element method, nanostructures, nanoporous gold, polypyrrole, epoxy resin, polyaniline, nanocomposite, numerical models

## Abstract

In this work, representative volume elements (RVEs) of composites, consisting of nanoporous gold and polymer, were investigated. Gold is of great interest as a special case of nanoporous metals as it deforms to large plastic strains when compressed, whereas normally nanomaterials allow only small deformations. The nanocomposite is modeled as a nanoporous monocrystal filled with a polymer. Different models of the phase behavior of nanoporous metal composites with the addition of a polymer component were studied. Three models of the mechanical behavior of gold were implemented: elasticity, elastic-plasticity, and the model of fracture with the degradation of properties. Three types of polymers were considered: polypyrrole (PPy), epoxy resin, and polyaniline (PANI), for which elasticity and elastic-plasticity models were implemented. The effect of the morphology of the nanocomposite on their mechanical response was numerically investigated using finite element analysis.

## 1. Introduction

Nanoporous materials are known as a type of three-dimensional porous solid with a characteristic nanoscale size. They have a morphology similar to macroscopic solid foams, but have smaller pore diameters and ligaments, as well as a high specific surface area. The interest in such a class of materials is also related to the possibility of creating nanocomposites on its basis by filling the empty space with another material in order to realize the necessary properties. The structure of nanoporous metals and composites based on them can be represented as a model of bicontinuous media, in which the components form interpenetrating scaffolds.

Nanoscale heterogeneous materials and, in particular, nanoporous materials and composites made of them are attracting growing attention of researchers due to their unique properties [1,2]. These materials have a specific coherent nanoscale structure with a continuous porous phase and can be created, for example, from different metals such as silver [3], platinum [4,5], copper [6], nickel [7], palladium [5,8,9], titanium [10], aluminium [11], and gold [4,5]. Recent studies have investigated the use of such metals as functional materials for catalysis, actuators, and probing [12,13].

Nanoporous gold has a bicontinuous network of nanoscale pores and solid ligaments [14]. Numerous experimental studies have shown that, in addition to relative density, the strength of nanoporous gold is strongly dependent on the average radius of the ligaments [15], while the macroscopic strength increases with the decreasing ligament size [16,17].

The prediction of the macroscopic properties and local response of the nanoporous material is complicated due to the inherent complex internal composition. For this purpose, mathematical modelling methods are applied to investigate the representative volume elements (RVEs) of the materials. To generate artificial samples of materials with a morphology similar to that observed experimentally, several methods were proposed [18,19,20].

Molecular dynamics studies were performed to quantify the evolution of the dislocations and configurations in a porous monocrystalline metal [21]. Modeling shows that nanovoids are important sources of dislocations. In uniaxial compression, dislocation shear loops originate on the pore surface. Plastic deformation occurs due to dislocation separations from the pore surfaces and the distribution of the dislocations in the ligaments, and the interaction of the dislocations at their contact with neighboring pores. As the voids begin to shrink, the density of the dislocations increases rapidly [22]. The results have shown that the dominant deformation mechanism of nanoporous metals is the bending of bonds at the joints of the structure, which is consistent with the experimental results in the compression.

The finite element method (FEM) is also used as a tool to investigate the mechanical properties of nanoporous media. FEM simulates the stiffness characteristics of porous solids with different configurations and relative densities [23,24,25]. Numerical modeling and analysis of the kinematics of the final deformation of ligaments, surface elasticity, and the initial stress effects of the porous medium have been performed [26,27]. To implement FEM modeling, mechanical properties (e.g., Young’s modulus, Poisson’s ratio, and yield strength) of the ligaments must be obtained by experiments or from other computational models of a smaller scale. In addition to three-dimensional finite elements, a series of works [14,28] have used beam finite elements bicontinuous media with interpenetrating phases.

The behavior of nanoporous gold has also been compared with the classical Gibson−Ashby scaling ratios for foams using finite element models [29,30]. According to published work in the field, when calculating the elastic properties numerically, both approaches produce results for yield values that exceed the Gibson−Ashby scaling relationship prediction for metal foams, and thus confirm the influence of other microstructural features besides porosity.

In the experiments with macroscopic stretching, nanoporous materials immediately show fragility. So far, the only way to prevent this is to create composites by introducing a polymer into the voids [31]. It has recently been shown that such a material can withstand significant deformation at a four-point bend [32]. The simulation of the mechanical behavior of nanocomposites based on foams is less common in the literature. A nanoporous metal polymer composite was modeled in [33] using a simplified two-dimensional RVE, investigating the plastic reaction and microstructure influence. The effective elastic properties of a realistic three-dimensional representative volume of such a nanocomposite were subsequently investigated [34,35].

At present, there are practically no hierarchical three-dimensional models of two-component nanoscale composites with a random structure that allow for reliable prediction of the deformation behavior of such structures depending on the physical factors and the stochastic nature of the structure morphology. Thus, modeling the behavior of whole RVE (on the scale of hundreds of nanometers) is essential in order to account for the mutual influence of the morphological structural elements.

The computational effort that would be required for the atomistic modeling of realistic RVE of nanoporous materials and nanoscale composites is often excessive. Consequently, continuum mechanics models numerically realized using the finite element method are used to study the dependence of the mechanical behavior on the morphology structure. In this case, the properties and constants required for the models can be obtained either from experimental studies or from molecular dynamics models. On the basis of finite element modeling, it is possible to investigate the influence of material morphological features on the mechanical behavior relatively quickly and efficiently. In particular, the processes of crack nucleation, crack propagation, and the final fracture of nanocomposites on the nanoscale can be studied.

## 2. Materials and Methods

### 2.1. Geometry Models

For the mechanics of heterogeneous media, an important task is to create three-dimensional models reflecting the morphological structure of real objects. The creation of geometric models of bicontinuous media simulating experimentally obtained images of the internal structure of nanoporous metals and nanocomposites, consisting of nanoporous metals and polymers, was performed based on space separation using the surface equation, which is given by setting the level for a random Gaussian field represented as a Fourier series [18,19,20]:

(1) $$f\left(x\right)=\frac{1}{\sqrt{N}}\sum_{i=1}^{N}c_{i}\cdot \cos\left(\frac{2\pi }{a}k_{i}\cdot x+\varphi_{i}\right),$$

where x is the position of the radius vector, a is the size of RVE, N is the number of harmonics, $\varphi_{i}$ is the wave phases that are evenly distributed on the interval [0,2π), and $k_{i}$ is the wave directions. The coefficient $c_{i}\;$is randomly selected. Different phases of a representative volume are determined by assigning points of space according to the following conditions: the point belongs to phase 1 if f(x)<ξ and phase 2 if f(x)≥ξ, where the parameter ξ defines the separation surface.

The approach was adapted to fit the internal structure of specific materials, such as nanoporous gold, as well as nanoporous gold-based polymer composites, by modifying the number of harmonics and wave parameters responsible for the stochasticity and regularity of the structures. For this purpose, images of material samples published in [14,36], obtained by scanning electron microscopy methods, were examined. 

**Figure 1:**
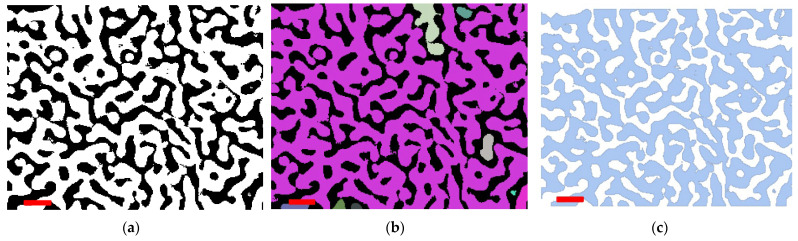
Some steps of the proposed image processing method: (**a**) threshold processing and morphological binarization of image; (**b**) morphologically connected components; (**c**) final representation as the discretized region. The red scale bar in the lower left corner represents 100 nm.

An example of a two-dimensional processed image of the nanoscale structure of nanoporous metals is shown in Figure 1. The original image was an electron photograph from [36] of the nanoporous gold obtained with a low-voltage field emission scanning electron microscope at 100,000 magnification and 5 kV voltage. Several image filters were applied in order to obtain a binarized two-component composition that is suitable for the morphological statistical analysis. A thresholding filter was used for filtering the parts of the image that were in the background (Figure 1a). In particular, it replaced values close to zero by zero using threshold specification. As the metallic phase was supposed to be continuous, morphological analysis was required to seek for disconnected parts (see Figure 1b, separated parts are highlighted). The thresholding parameter was then tuned to minimize the number of separated regions (0.6 in the considered case). Finally, analytical binarized region was formed for further morphological analysis.

An algorithm has been created and implemented in Wolfram Mathematica that allows for analyzing two-dimensional experimentally obtained black-and-white images of the structure of nanoporous materials using the tools of mathematical morphology. The goal of this algorithm was to find the parameters that are required to construct equivalent three-dimensional RVEs of nanoscale structures. Physical and statistical morphological characteristics were used as comparison parameters in order to find correspondence between two-dimensional images and three-dimensional models [37,38,39,40,41]. The volume fraction and the perimeter of the interface between the two phases (the surface area of the interface in the three-dimensional case) were used as the physical characteristics, and the correlation functions were used as the statistical ones. In the general case, the *n*-th order correlation function is defined via a random indicator function, as follows:

(2) $$K_{\alpha }^{\left(n\right)}({\vec{r}}_{1},\ldots ,{\vec{r}}_{n})=\langle \int_{V_{1}}\int_{V_{2}}\ldots \int_{V_{n}}\left(\lambda_{\alpha }\left({\vec{r}}_{1}\right)-p\right)\left(\lambda_{\alpha }\left({\vec{r}}_{2}\right)-p\right)\ldots \left(\lambda_{\alpha }\left({\vec{r}}_{n}\right)-p\right)dV_{1}dV_{2}\ldots dV_{n}\rangle ,$$

where $\lambda_{\alpha }\left(\vec{r}\right)$ is the random indicator function for aphase α, which can take two values: 1 if a radius-vector $\vec{r}$ points at the phase α, and 0 otherwise; p is the volume fraction of the phase α. The correlation functions depend only on the distance between the radius vectors $\left|{\vec{r}}_{i}-{\vec{r}}_{n}\right|$. In this work, the second-order correlation functions were used for the comparison of 2D images and 3D RVEs. Using this comparative algorithm, geometric models of three-dimensional RVEs of bicontinuous heterogeneous media, reflecting the structure of the samples on the images, have been iteratively obtained. The set of parameters included the volume fraction of phases (was controlled by changing the level parameter ξ for a random Gaussian field), regularity of structures (wave parameters and number of harmonics N in the Fourier series), size of internal structural components (scale parameters in the Fourier series), and size of RVE. These parameters were calibrated according to the proposed algorithm by iterative comparison and optimization of the physical and statistical descriptors until the required tolerance was reached.

**Figure 2:**
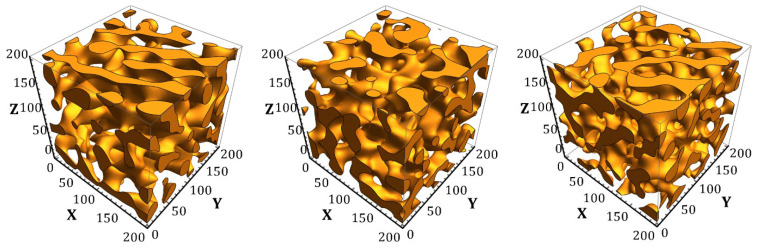
Three-dimensional RVEs of porous two-phase random structure of nanoporous metals obtained from a two-dimensional image using the proposed restoration algorithm (scale in nm).

Using the proposed image processing method, three-dimensional RVEs of nanoporous metals with dimensions of 200 nm × 200 nm × 200 nm in size containing random combinations of ligaments were created. The structures with the following polymer inclusion volume fractions were investigated: *p* = 0.664, *p* = 0.665, *p* = 0.678, *p* = 0.680, *p* = 0.696. Examples are shown in Figure 2.

**Figure 3:**
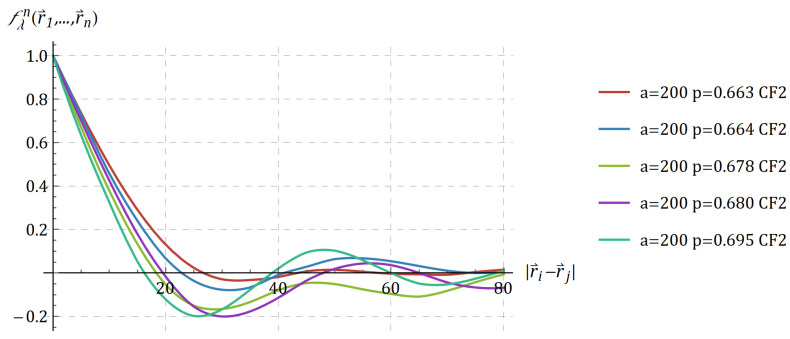
Second-order correlation functions for three-dimensional RVEs of nanoporous gold with different volume fractions *p*.

Examples of the second-order correlation functions are presented in Figure 3 for the structures with different volume fractions.

To create finite element models of the derived RVEs, the algorithm that discretized the region with four-point tetrahedral elements was developed using the extended capabilities of the Wolfram Mathematica software. The surface of a geometric region was discretized into triangles with a fixed maximum value of the longitudinal dimension. This two-dimensional sampling was then used as the basis for the creation of a tetrahedral mesh. The size of the mesh elements was controlled, as well as the occurrence of possible mesh defects, such as extremely small components, singular vertices, and faces of tetrahedrons with a near-zero area. 

**Figure 4:**
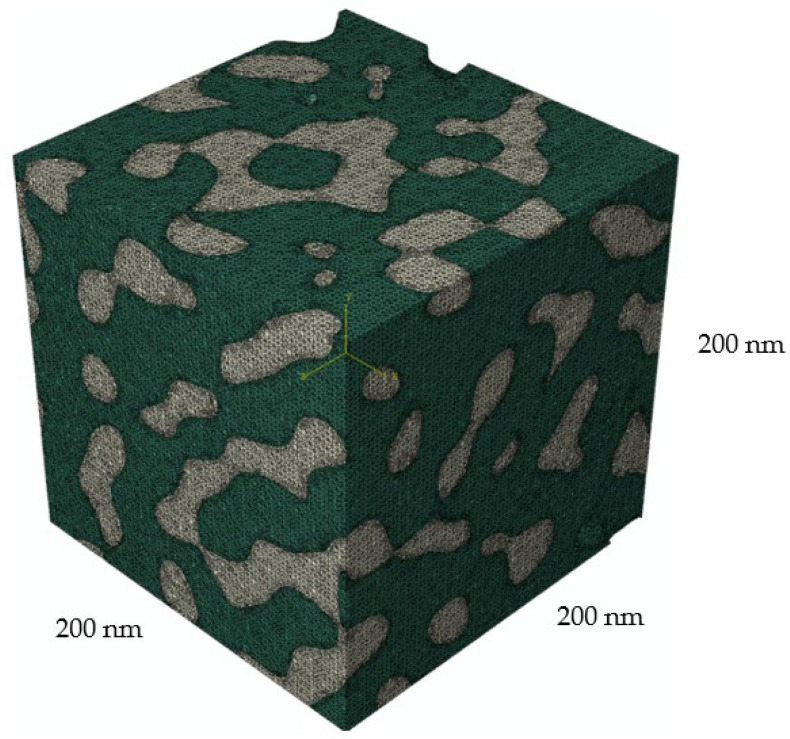
Two-phase finite-element model with a medium-density tetrahedral mesh.

Figure 4 shows an example of the generated finite-element model of a two-phase bicontinuous structure. White represents the gold phase, green is the filler (polymer) phase. The SIMULIA Abaqus software was used for the finite element modelling. The characteristics of this numerical realization: number of elements in the inclusion phase 1,237,405, number of elements in matrix phase 939,689, total elements 2,177,094, number of nodes 482,076, and the maximum element size ratio is 0.06 for the side size of RVE. 

### 2.2. Material Models

**Figure 5:**
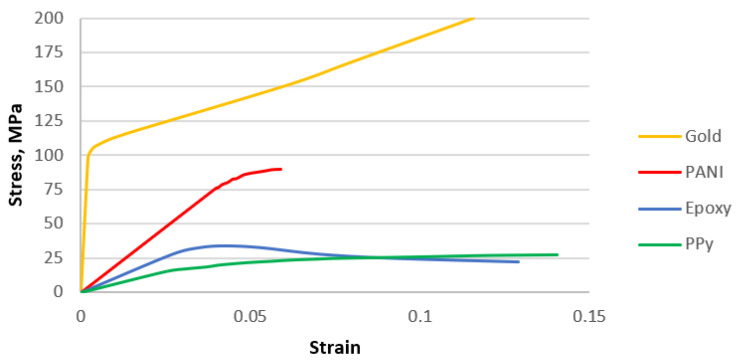
Plastic behavior of the constituents.

Various mechanical models of the of the components of the studied nanocomposites were investigated. Thus, for RVEs of the nanocomposite material, calculations were performed in which the gold phase was considered elastic, elastoplastic, and elastoplastic with possible damage accumulation. Several types of the polymeric phase were considered as a filler, which was modelled as an elastic and elastoplastic material. The physical and mechanical properties of the phases were obtained from experimental works [17,42,43], and they were also confirmed by some numerical studies based on molecular dynamics methods [33,44,45]. The properties of the constituents for the gold-polymer nanocomposites are presented in Table 1 and Figure 5.

A special custom UMAT subroutine for SIMULIA Abaqus was used to implement the model of fracture with the degradation of properties. This model was introduced for the gold phase, the stiffness tensor of which additionally contained the internal state variables D (see Figure 3). These variables are so-called degradation coefficients and characterize the occurrence of structural failure. At the initial moment of time, all coefficients are equal to zero, thus the material model does not differ from the usual one.

(3) S(D)=[1E−νE−νE0 0 0         1E(1−D)−νE0 0 0          1E0 0 0    1μ00 sym.   1μ0      1μ],

However, when the degradation criterion is fulfilled, the coefficients become equal to some constant (in a range from 0 to 1), and lowered the values of the components of the stiffness tensor. In this work, the degradation coefficient D=0.9 was chosen; it affected only the $S_{22}$ component of the compliance tensor when the criterion for maximum principal stresses was fulfilled by principal stress along the vertical axis, i.e., $\sigma_{2}\geq \sigma_{s}$, where $\sigma_{s}$ is the critical stress value. The field of degradation criterion fulfillment varied from 0 to 0.9. The value of the field “0.9” shows the places where elastic properties were reduced. These can be used to qualitatively estimate the areas of possible failure. Similar approaches using custom subroutines have been actively implemented recently, for example in [52,53].

### 2.3. Boundary Conditions

**Figure 6:**
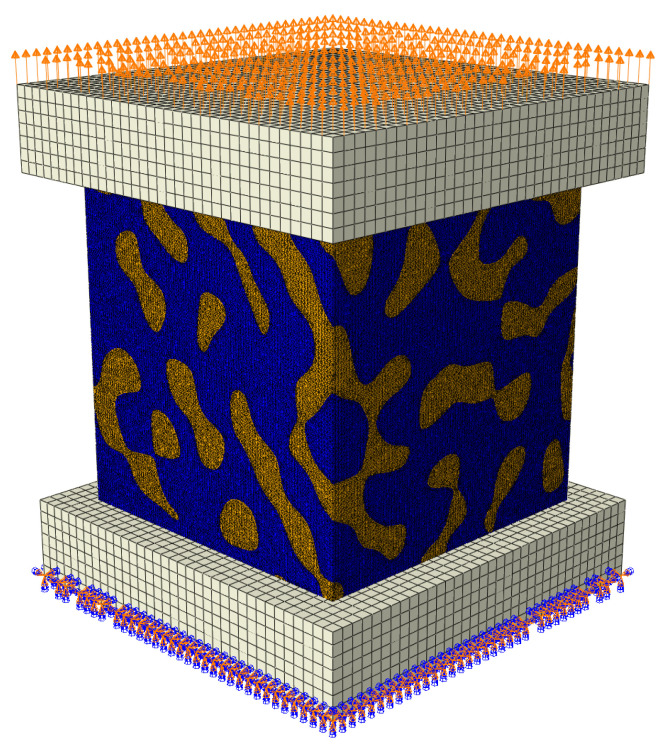
Scheme of the specimen loading.

The RVEs were subjected to tensile and compressive loads applied through loading plates. Those support loading plates were bonded to the top and bottom surfaces of the RVEs with frictionless contact conditions specified (Figure 6). The properties of support loading plates were much more rigid than the constituents of the RVEs. The load was applied in displacements of u=2 nm along the vertical axis. The ideal contact was assumed between the phases.

## 3. Results

The deformation diagrams obtained for the variation of constituent properties were built for the RVEs created with different volume fractions and internal morphologies. The distributions of stress and strain fields in the RVEs as an effective media and in their individual components were investigated. The processes of the formation of stress concentrators for different models of mechanical behavior of the phases were studied.

The following results for the numerical models of nanocomposites can be divided into subgroups according to the following criteria: by gold phase material model, by polymer phase material mechanical model, by the polymer phase material itself, by geometry model, by volume fraction of nanocomposite inclusions, and by loading model. In this paragraph, force−displacement plots will be demonstrated for two geometry models with volume fractions of 0.680 and 0.664.

This paragraph is divided into two subparagraphs. The first subparagraph is devoted to numerical models where the polymer phase is an elastic material. The second one is devoted to numerical models where the polymer phase is an elastoplastic material. Each subparagraph will describe all three polymeric materials: polypyrrole (PPy), epoxy resin, and polyaniline (PANI).

### 3.1. Nanoporous Gold with Elastic Polymer

**Figure 7:**
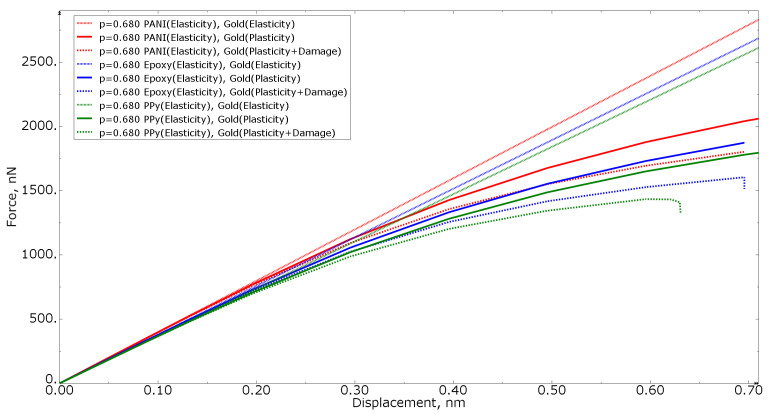
Tensile force−displacement curve for numerical models of the same polymer inclusion volume fraction at different gold material models.

Figure 7 shows the force−displacement curves for tension loading of numerical models with the same polymer volume fraction of 0.680 for all three polymer materials, as well as with different gold material models (elastic formulation, elastoplastic formulation, and elastoplastic formulation with property degradation procedure). The higher the stiffness of the nanocomposite polymer, the higher the curve in the graph. The more compliant the gold phase material, the lower the curve on the graph.

**Figure 8:**
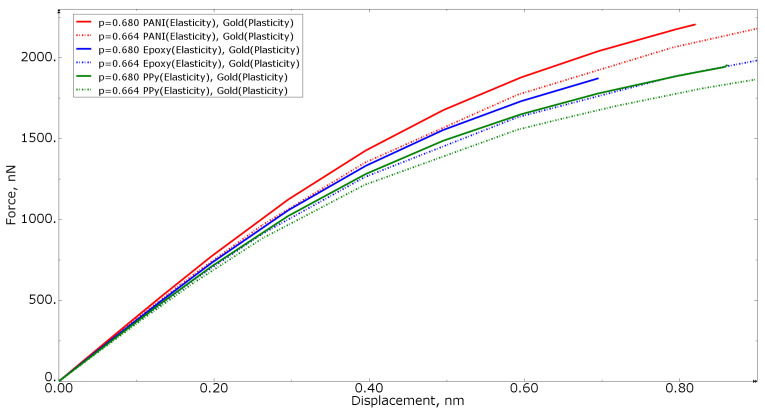
Tensile force−displacement curve for numerical models of elastoplastic gold material models at different polymer inclusion volume fractions.

Figure 8 shows the tension force−displacement curves for numerical models with elastoplastic gold material models, but different polymer inclusion volume fractions of 0.680 and 0.664 for all three polymer materials. For simple cases, the higher the volume fraction of the more compliant phase, the more compliant the entire composite is. In Figure 8, this condition is not satisfied: the curves of the nanocomposite with a polymer inclusion volume fraction of 0.680 are higher than the nanocomposite with a volume fraction of 0.664. This allows for concluding that not only the volume fraction but also the morphology of the nanocomposite inclusions makes a difference: even for RVEs with the approximately same volume fraction, the results can differ due to the randomness of morphological composition.

**Figure 9:**
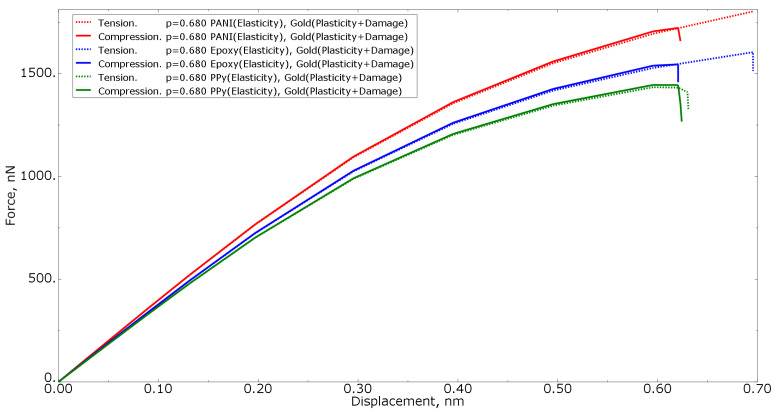
Force−displacement curve for numerical models of elastoplastic gold material models with the procedure of degradation of elastic properties at the same polymer inclusions volume fraction and different loading models.

Figure 9 shows the tension force−displacement curves for numerical models with the same polymer inclusion volume fraction of 0.680 for all three polymer materials, as well as with different loading conditions (tension/compression). From the curves, it can be concluded that the structure will collapse at lower strain values under compression loading than when subjected to tension.

**Figure 10:**
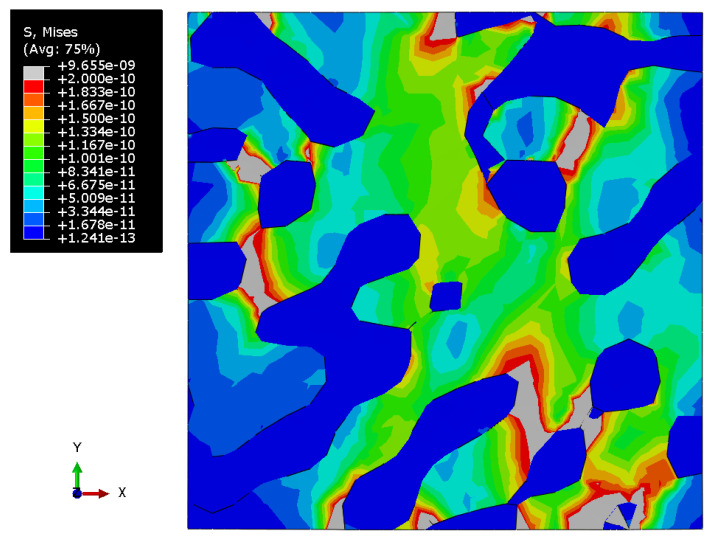
Tensile Mises stress fields (Pa) of the bicontinuous structure with a polymer inclusions volume fraction p=0.680, which has the elastic properties of the gold phase and elastic properties of the polymer.

**Figure 11:**
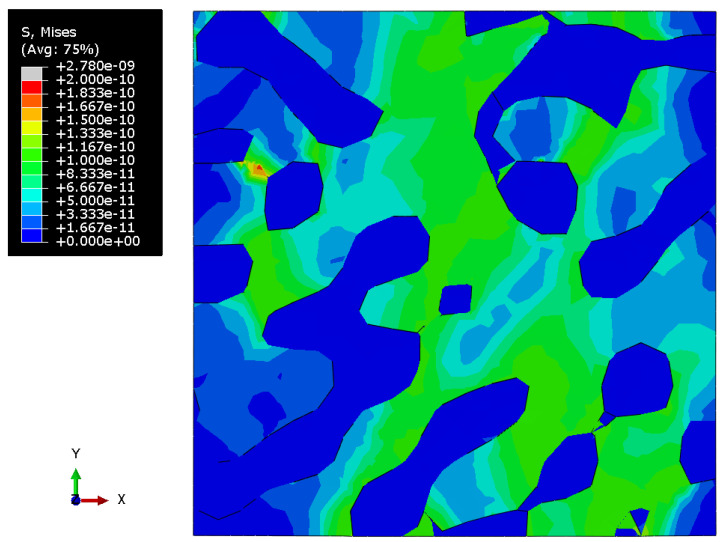
Tensile Mises stress fields (Pa) of the bicontinuous structure with a polymer inclusions volume fraction p=0.680 with elastoplastic properties of the gold phase and elastic properties of the polymer.

**Figure 12:**
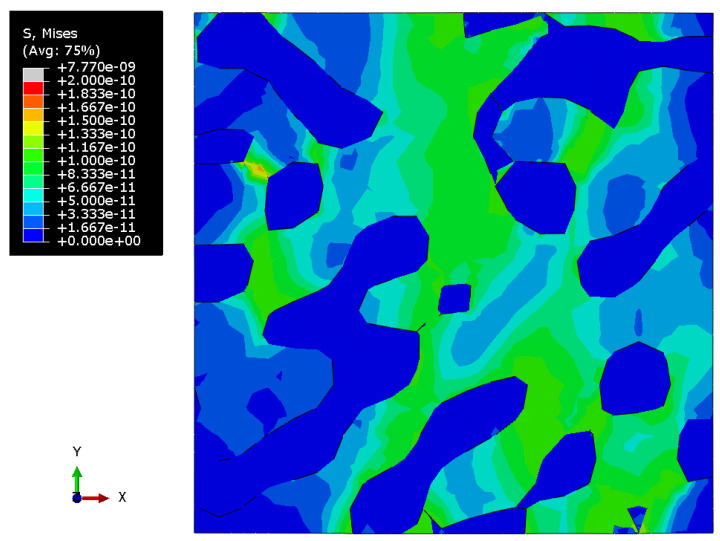
Tensile Mises stress fields (Pa) of the bicontinuous structure with polymer inclusions volume fraction p=0.680 with elastic properties of the polymer and elastoplastic properties of the gold phase with the procedure of degradation of elastic properties.

Figure 10, Figure 11 and Figure 12 show the Mises stress fields (Pa) in the RVE of a nanocomposite with a polymer inclusion volume fraction of 0.680 at the same moment of loading. Figure 10 shows the Mises stress field in the elastic case of the gold phase. Figure 11 represents the stress field in the elastoplastic case of the gold phase. Figure 12 shows stress field in elastoplastic case of the gold phase with the property degradation procedure.

**Figure 13:**
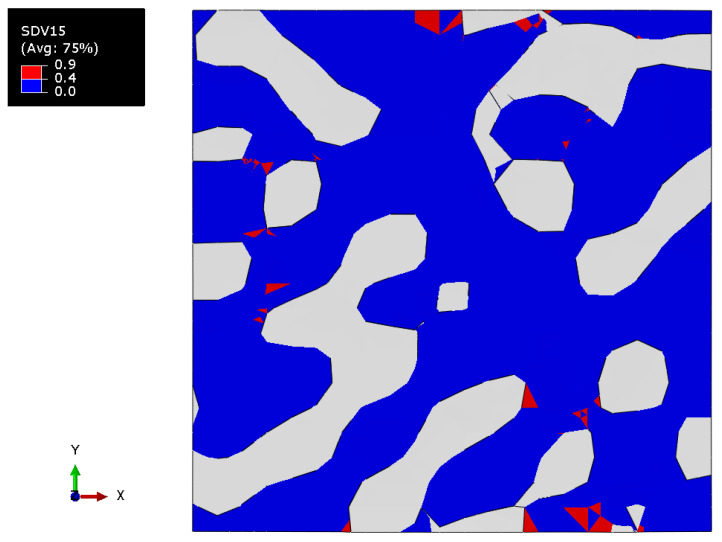
The tensile degradation criterion field of the nanocomposite with a polymer inclusions volume fraction p=0.680 with elastic properties of the polymer and elastoplastic properties of the gold phase with the procedure of the degradation of elastic properties.

Figure 13 shows the field of the criterion of degradation of properties (dimensionless unit) for a nanocomposite with a polymer phase volume fraction of 0.680 at the elastoplastic formulation of the gold phase with the property degradation procedure. The value of the field “0.9” indicates the regions where the criterion is fulfilled.

### 3.2. Nanoporous Gold with Elastoplastic Polymer

**Figure 14:**
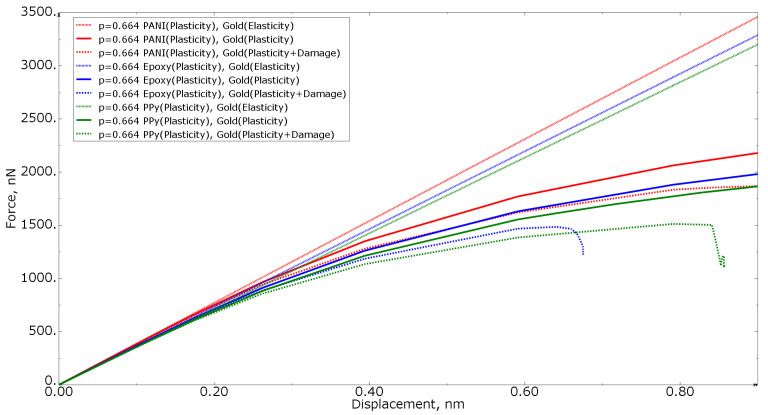
Tensile force−displacement curve for numerical models of the same polymer inclusion volume fraction at different gold material models.

Figure 14 shows the force−displacement curves in tension for numerical models with the same polymer volume fraction of 0.664 for all three polymer materials, as well as with different gold material models (elastic formulation, elastoplastic formulation, and elastoplastic formulation with a property degradation procedure).

**Figure 15:**
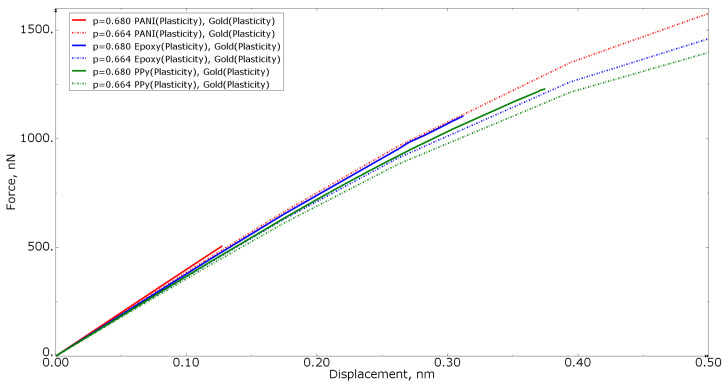
Tensile force−displacement curve for numerical models of elastoplastic gold material models at different polymer inclusion volume fractions.

Figure 15 shows the tension force−displacement curves for numerical models with an elastoplastic gold material, but different polymer inclusion volume fractions of 0.680 and 0.664 for all three polymer materials. When analyzing Figure 15, a similar conclusion can be made as for Figure 8—the curves of the nanocomposite with a polymer volume fraction of 0.680 are higher than the nanocomposite with a polymer volume fraction of 0.664, indicating the influence of the morphology of the nanocomposite inclusions.

**Figure 16:**
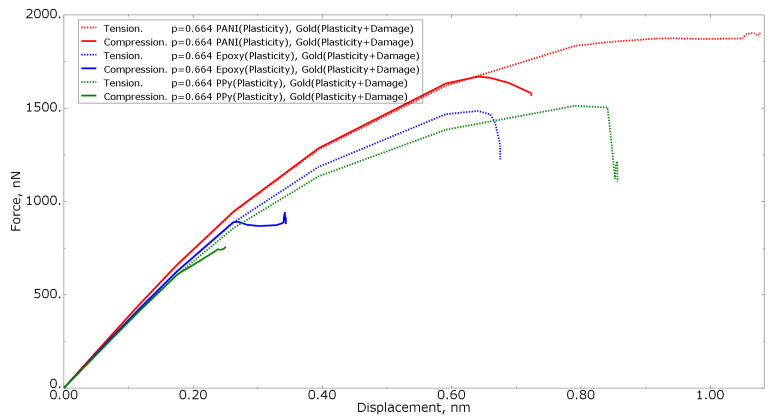
Force−displacement curve for numerical models of elastoplastic gold material models with the procedure of the degradation of elastic properties at the same polymer inclusions volume fraction and different loading models.

Figure 16 shows the tension force−displacement curves for numerical models with the same polymer inclusion volume fraction of 0.664 for all three polymer materials subjected to different loading conditions (tension/compression). It can be concluded that, due to the complexity of the geometry, the structure will collapse faster in compression than in tension. These results for equal RVE models also show the influence of the filler properties on the mechanical behavior of the material.

**Figure 17:**
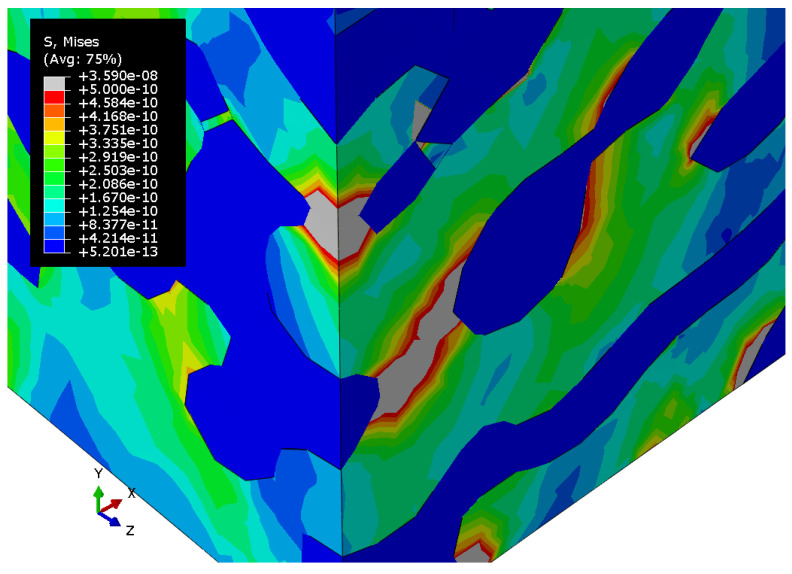
Tensile Mises stress fields (Pa) of a nanocomposite with a polymer volume fraction p=0.664, which has the elastic properties of the gold phase and elastoplastic properties of the polymer.

**Figure 18:**
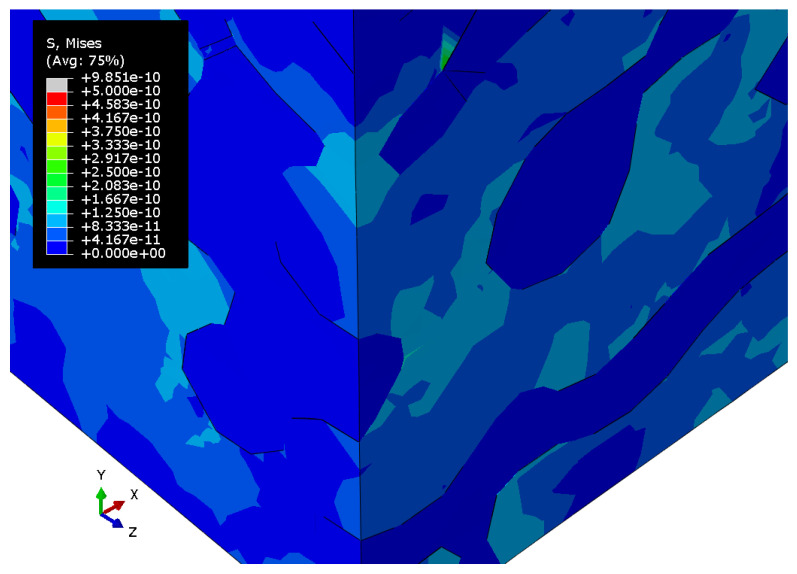
Tensile Mises stress fields (Pa) of a nanocomposite with polymer volume fraction p=0.664 with the elastoplastic properties of the gold phase and the elastoplastic properties of the polymer.

**Figure 19:**
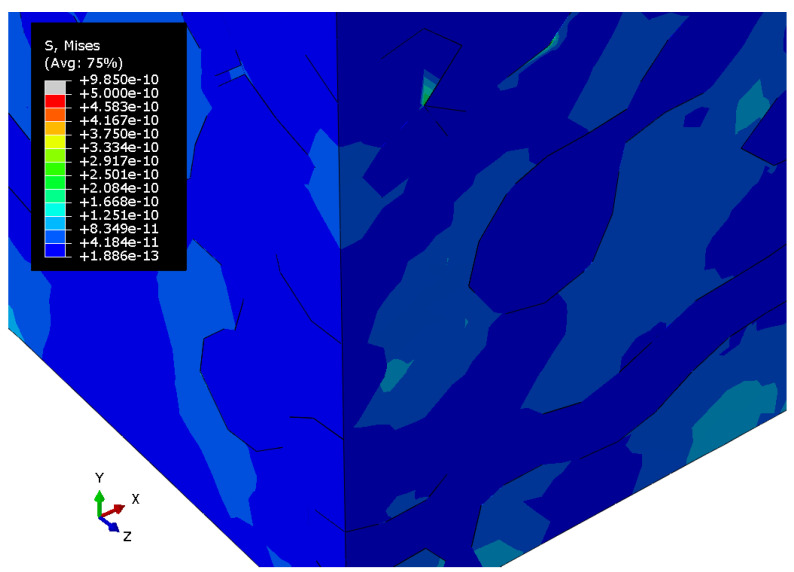
Tensile Mises stress field (Pa) of a nanocomposite with a polymer volume fraction p=0.664 with elastoplastic properties of the polymer and elastoplastic properties of the gold phase with the procedure of the degradation of elastic properties.

Figure 17, Figure 18 and Figure 19 show the Mises stress fields (Pa) in the RVE of a nanocomposite with a polymer inclusion volume fraction of 0.664 at the same moment of loading. Figure 17 shows the stress field in the elastic case of the gold phase. Figure 18 represents the stress field in the elastoplastic case of the gold phase. Figure 19 shows the stress field in the elastoplastic case of the gold phase with the property degradation procedure.

**Figure 20:**
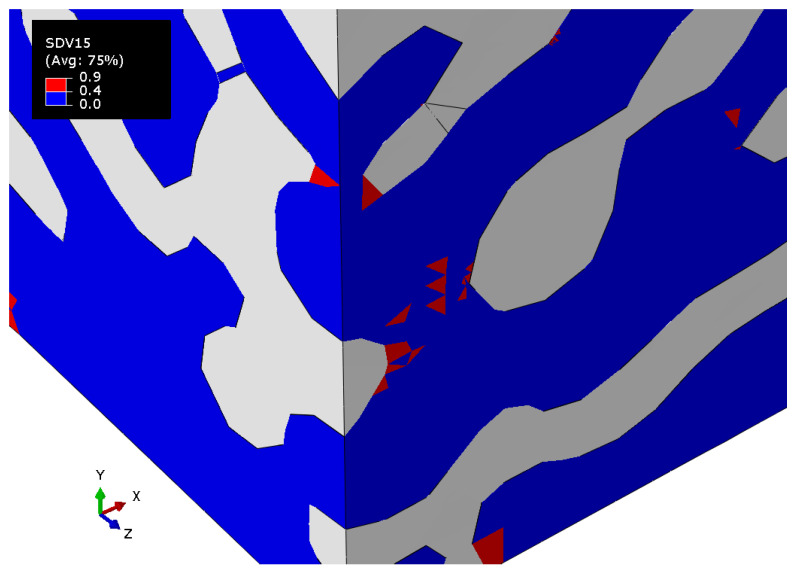
Tensile degradation criterion field of a bicontinuous structure with a polymer inclusions volume fraction of p=0.664 with elastoplastic properties of the polymer and elastoplastic properties of the gold phase with the procedure of the degradation of elastic properties.

Figure 20 shows the field of the criterion of the degradation of properties (dimensionless unit) of a nanocomposite with a polymer volume fraction of 0.664 with elastoplastic formulation of gold phase including the property degradation procedure. The value of the field “0.9” indicates the regions where the criterion was met.

## 4. Conclusions

Deformation diagrams were numerically calculated for the RVEs of nanocomposites with different volume fractions and internal morphologies, taking into account the variation of the phase property mechanical models. The distributions of stress and strain fields in the RVEs and in its individual components were investigated using finite element numerical modelling. The processes of the formation of stress concentrators for different models of the mechanical behavior of the constituents were studied.

The influence of the properties of the filler phase in gold-polymer nanocomposites on the mechanical behavior of the morphologically authentic RVEs was demonstrated. A comparative analysis of the constituents’ properties combinations was performed based on the results of several considered case studies. The effect of the filler volume fraction was studied. It has been established that the mechanical properties of nanocomposites are significantly affected by their internal composition (composition, size of ligaments, etc.). With the growth of filler volume fractions, the maximum achieved stress increases, and the displacements at failure decrease with increasing the gold volume fraction. The data obtained show a ligament size-dependent behavior of the two-phase gold-polymer composite. Composite samples with a smaller ligament size fractured at lower strain values than the samples with larger ligaments, and also exhibited a broader scatter of yield strength. Nanocomposites with a polymer component exhibited a close to linear relationship for the maximum load at failure when increasing the volume fraction of the gold phase. The obtained relations demonstrate the possibility of using continuum mechanics methods to model the behavior of nanoscale materials.

## Figures and Tables

**Table 1:** Material parameters for gold matrix and polymer filler.

Parameter	Symbol	Value	Ref.
Gold			
Young’s module	E	48,000 MPa	[33,46]
Poisson’s ratio	ν	0.44	[33,46]
Elastic limit	$\sigma_{y}$	96 MPa	[33]
Ultimate strength	$\sigma_{s}$	190 MPa	[47]
Stress−strain curve	-	-	[33]
Polypyrrole (PPy)			
Young’s module	E	600 MPa	[31]
Poisson’s ratio	ν	0.3	[31]
Elastic limit	$\sigma_{y}$	15 MPa	[48]
Stress−strain curve	-	-	[48]
Epoxy resin			
Young’s module	E	1038 MPa	[33,49]
Poisson’s ratio	ν	0.35	[33,49]
Elastic limit	$\sigma_{y}$	28 MPa	[33,49]
Stress−strain curve	-	-	[33,49]
Polyaniline (PANI)			
Young’s module	E	1910 MPa	[50]
Poisson’s ratio	ν	0.38	[51]
Elastic limit	$\sigma_{y}$	76 MPa	[50]
Stress−strain curve	-	-	[50]
